# Supplementary material for: Pharmacological modulation of developmental and synaptic phenotypes in human SHANK3 deficient stem cell-derived neuronal models
Source: Transl Psychiatry. 2024 Jun 10;14:249. doi: 10.1038/s41398-024-02947-3 (PMC11165012; doi:10.1038/s41398-024-02947-3)
Supplement: Supplementary file 1 — Supplementary information [file 41398_2024_2947_MOESM1_ESM.docx]

# Supplementary figures

**Figure S1 referring to Figure 1:** Quality control data of (A) healthy control and (B) PMDS patient-derived iPSC lines. Fluorescence in situ hybridization (FISH) was used to detect chromosome aberrations. Molecular karyotyping was performed analyzing single nucleotide polymorphisms (SNP). Fluorescence-activated cell sorting (FACS) was used to determine the expression of Stage specific embryonic antigen 4 (SSEA-4), a marker for cells with pluripotent and stem cell-like characteristics.

**Figure S2 referring to Figure 1:** Exposure to CRISPR-Cas9 did not alter the karyotype or stem cell properties of SA001 cell line-derived clonal populations. Fluorescence in situ hybridization (FISH) was used to detect chromosome aberrations. Fluorescence-activated cell sorting (FACS) was used to determine the expression of Stage specific embryonic antigen 3 and 4 (SSEA-3 and SSEA-4), a marker for cells with pluripotent and stem cell-like characteristics.

**Figure S3 referring to Figure 1:** SHANK3 expression and proliferation is reduced in CRISPR-modified NPCs. (A) Representative images of Western blots with and without SHANK3 peptide blocking of WT2 and HT3 NPCs lysed around D30 and quantification of the Western blot SHANK3 signal relative to the GAPDH signal using the Area Under the Curve (AUC) method in FIJI/ImageJ. (B) RT-qPCR was performed using Taqman probes against SHANK3 on D30. The expression level was determined relative to PPIA. At least two independent experiments were performed for Western blot and RT-qPCR experiments. (C) Nuclei count and EdU (5-ethynyl-2'-deoxyuridine) incorporation assay for all unmodified and SHANK3 heterozygous mutation Cas9-exposed clones for D6, D9 and D14 of differentiation. The mean of at least three technical replicates is shown with the 95% confidence interval. (D) SOX2-postive cell ratio for all unmodified and SHANK3 heterozygous mutation Cas9-exposed clones on D6 of differentiation. (E) ß-tubulin III (TUJ1) quantification for all unmodified and SHANK3 heterozygous mutation Cas9-exposed clones on D6 of differentiation. The boxplots show the median and the 2^nd^ and 3^rd^ quartiles of at least eight technical replicates from a single differentiation. Whiskers indicate 1.5X of the interquartile range.

**Figure S4 referring to Figure 1:** Lower proliferation and higher neuronal differentiation in SHANK-deficienct NPCs (pooled data from Figure 2). (A) Immunostaining-based quantification of the ratio of proliferating and differentiating healthy donor (blues) and patient-derived NPCs (reds). (B) Same as (A), but in CRISPR-engineered isogenic NPC pairs. Each small datapoint represents technical replicate data from one 384-well plate well. Larger markers represent the medians of all cell lines or clones of independent differentiation experiments (N=3). The boxplots show the median and the 2nd and 3rd quartiles of the data. Whiskers indicate 1.5X of the interquartile range. Welch's unequal variances t-test was performed using the medians of independent differentiation experiments. ns = not significant, * = p < 0.05, ** = p < 0.01, *** = p < 0.001.

**Figure S5 referring to Figure 2:** Synaptic SHANK3 levels are lower in SHANK3-deficent neurons. (A) The PMDS patient-derived ASD04, healthy control PC056, and CRISPR-engineered SHANK3-deficient HT3 and isogenic control WT2 NPC lines were differentiated for 30 days and fluorescently stained. Using quantitative image analysis, in Synapsin 1/SHANK3 double positive synapses located in the MAP2 network the mean SHANK3 signal intensity was quantified. Each datapoint represents technical replicate data from one 384-well plate well. Larger markers represent the medians of independent differentiation experiments. The boxplots show the median and the 2^nd^ and 3^rd^ quartiles of the data. Whiskers indicate 1.5X of the interquartile range. Welch's unequal variances t-test was performed using the medians of independent differentiation experiments. ns = not significant, * = p < 0.05.

**Figure S6 referring to Figure 4:** Quality assessment of small molecules screening in CRISPR-engineered NPCs. (A) Ratios of HuC/D and Ki67 positive WT2 and HT3 cells across screening plates. Cells were treated with neutral control DMSO or positive proliferation-inducing control compounds HA1077 (WT2) or GSK-25 (HT3). (B) Z’ factor per plate calculated between chemical controls DMSO and GSK-25. (C) Relationship of HuC/D and Ki67 positivity ratio of compound- and DMSO-treated wells for both replicates. The Pearson correlation coefficients are indicated.

**Figure S7 referring to Figure 4:** Validation of SHANK3-specific hyperdifferentiation rescuing chemical compounds in PMDS patient and healthy control-derived NPCs. (A) Representative images of healthy control NPC line PC056 and PMDS patient cell line ASD04 stained against proliferation marker Ki67 and differentiation marker HuC/D and treated with DMSO or Benproperine. (B) Dose-response effects of 6 compounds on the ratio of differentiating HuC/D positive ASD04 and control PC056 NPCs. The shaded area in the line graphs corresponds to the 95% confidence interval of triplicate experiments.

**Figure S8 referring to Figure 5:** Identified compounds require residual SHANK3 levels for activity. (A) Nuclei count, and ratios of Ki67 and HuC/D positive cells in knockout SHANK3-/- D6 NPCs. The dashed line indicates the DMSO baseline. The shaded area in the line graphs corresponds to the 95% confidence interval. (B) RT-qPCR was performed using Taqman probes against annotated compound targets DRD2, HRH1, and ARPC2 on D6. The expression level was determined relative to PPIA. The individual datapoints of three independent experiments are shown. The bars indicate the median value.

**Figure S9 referring to Figure 6:** Identified compounds impact synapse formation. (A) HT3 mutant or WT2 isogenic control cells were treated with Alimemazine and Boldine early (D1-3) during differentiation and synapses were quantified after 35 days of differentiation. The boxplots show the median and the 2^nd^ and 3^rd^ quartiles of the data. Whiskers indicate 1.5X of the interquartile range. Welch's unequal variances t-test was performed. ns = not significant, * = p < 0.05, ** = p < 0.01, *** = p < 0.001.

**Figure S10 referring to the Material & Methods section:** Image analysis. (A) Schematic depiction of image segmentation to determine Ki67, EdU or HuC/D positive cells. (B) Schematic depiction of image segmentation for neurite network, and pre- and post-synapse detection. Only a triple positive signal (pre-, and post-synapse on neurite) within the size threshold was considered a synapse. (C) Screen capture of the used PhenoLink software for image analysis. Two example plugins are shown for the detection of proliferating or differentiating cells (top panel) and synapse detection (bottom panel).

# Supplementary tables

**Table S1:** SHANK3-deficiency specific and dose-response confirmed chemical compounds leading to hyperproliferation and hypodifferentiation in CRISPR-engineered NPCs.

**Table S2:** Key resource table.

**Table S3:** Primary screen hits in CRISPR SHANK3+/- neuronal progenitor cell line HT3.

**Table S4:** Used cell lines.
