# Supplementary material for: Pharmacological modulation of developmental and synaptic phenotypes in human SHANK3 deficient stem cell-derived neuronal models
Source: Transl Psychiatry. 2024 Jun 10;14:249. doi: 10.1038/s41398-024-02947-3 (PMC11165012; doi:10.1038/s41398-024-02947-3)
Supplement: Supplementary file 2 — Table S1 [file 41398_2024_2947_MOESM2_ESM.docx]

| Compound name | CAS number | Target | Detailed annotation (Selleckchem) | SHANK3 specificity in CRISPR NPCs | SHANK3 specificity in patient NPCs |
| --- | --- | --- | --- | --- | --- |
| Alimemazine Tartrate | 4330-99-8 | Unknown | Phenothiazine derivative that is used as an antipruritic. | ++  (toxic at high conc. in control) | ++ |
| Benproperine phosphate | 19428-14-9 | Actin-related protein 2/3 complex subunit 2 (ARPC2) | Cough suppressant. Is an orally active, potent actin-related protein 2/3 complex subunit 2 (ARPC2) inhibitor. Attenuates actin polymerization. | ++  (toxic at high conc. in control) | ++ |
| Boldine | 476-70-0 | Unknown | Isolated from *Peumus boldus*, has alpha-adrenergic antagonistic properties. Farnesoid X receptor (FXR) agonistic properties. | +++  (toxic at high conc. in control) | + |
