# Supplementary material for: Pharmacological modulation of developmental and synaptic phenotypes in human SHANK3 deficient stem cell-derived neuronal models
Source: Transl Psychiatry. 2024 Jun 10;14:249. doi: 10.1038/s41398-024-02947-3 (PMC11165012; doi:10.1038/s41398-024-02947-3)
Supplement: Supplementary file 3 — Table S2 [file 41398_2024_2947_MOESM3_ESM.docx]

| REAGENT | SOURCE | IDENTIFIER | DILUTION |
| --- | --- | --- | --- |
| Neuronal progenitor cell generation |  |  |  |
| Noggin | Peprotech | 120-10C | 500 ng/ml |
| SB431542 | Tocris | 1614 | 20µM |
| EGF | Peprotech | AF-100-15 | 10ng/ml |
| FGF-2 | Peprotech | 100-18B | 10ng/ml |
| BDNF | Peprotech | 450-02 | 20ng/ml |
| StemMACS medium | Miltenyi Biotec | 130-132-985 |  |
| CloneR Supplement | StemCell Technologies | 05888 |  |
| Labware |  |  |  |
| 384-well plates | Perkin Elmer | 6007558 |  |
| CytoView MEA plates | Axion Biosystems | M768-tMEA-48W |  |
| Coating | | | |
| Polyornithine | Sigma | P4957-50 mL | 1:6 |
| Polyethyleneimine | Sigma | 408727 | 1:1000 |
|  |  |  |  |
| DPBS | Gibco | 14190-136 | 1 |
| Laminin | Invitrogen | 23017-015 | 1:500 / 1:100 (for MEA) |
| Basal medium | | | |
| DMEM | Gibco | 31331-028 | 1:2 |
| Neurobasal | Gibco | 21103-049 | 1:2 |
| ß-mercaptoethanol | Gibco | 31350-010 | 1:1000 |
| Penicillin-Streptomycin | Gibco | 15140-122 | 1:1000 |
| Proliferation medium | | | |
| Basal medium |  |  | 1 |
| N2 | Gibco | 17502-048 | 1:100 |
| B27 | Gibco | 12587-010 | 2:100 |
| EGF | Peprotech | AF-100-15 | 1:1000 |
| FGF2 | Peprotech | 100-18B | 1:1000 |
| BDNF | Peprotech | 450-02 | 1:1000 |
| Differentiation medium | | | |
| Basal medium |  |  | 1 |
| N2 | Gibco | 17502-048 | 1:100 |
| B27 | Gibco | 12587-010 | 2:100 |
| BrainPhys-based medium | | | |
| BrainPhys Neurobasal Medium | StemCell Technologies | 05790 | 1 |
| Neurocult SM1 supplement | StemCell Technologies | 05711 | 1:50 |
| N2 Supplement-A | StemCell Technologies | 07152 | 1:100 |
| BDNF | StemCell Technologies | 78005 | 1:5 000 |
| GDNF | StemCell Technologies | 78058 | 1:5000 |
| dbcAMP | StemCell Technologies | 73886 | 1:200 |
| L-Ascorbic Acid | Sigma | A4403 | 1:10000 |
| Western blotting |  |  |  |
| NuPAGE LDS Sample Buffer | ThermoFisher | NP0007 | 1:4 |
| NUPAGE Novex 3-8% Tris-acetate gel | ThermoFisher | EA03752BOX |  |
| NUPAGE Tris-acetate SDS running buffer | ThermoFisher | LA0041 | 1 |
| PVDF membrane | Biorad | 1620177 |  |
| Skim milk powder | ThermoFisher | LP0031B | 5% |
| SuperSignal West Dura | ThermoFisher | 34075 | 1 |
| SHANK3 blocking peptide | MyBioSource | MBS152000 | 1 μg/ml |
| RT-qPCR | | | |
| SHANK3 primer (FAM labelled) | ThermoFisher | Hs01393533_m1 |  |
| GAPDH primer (VIC labelled) | ThermoFisher | Hs02758991_g1 |  |
| PPIA primer (VIC labelled) | ThermoFisher | Hs03045993_gH |  |
| DRD2 primer (FAM labelled) | ThermoFisher | Hs00241436_m1 |  |
| HRH1 primer (FAM labelled) | ThermoFisher | Hs00911670_s1 |  |
| ARPC2 primer (FAM labelled) | ThermoFisher | Hs01031740_m1 |  |
| Fast Advanced Cells-to-CT TaqMan kit | ThermoFisher | A35374 |  |
| Compound treatment | | | |
| DMSO | Sigma | D2650 | 0.1% |
| GSK-25 | MedChemExpress | HY-14362 | 1 µM |
| HA-1077 | Sigma | 371970-1mg | 10 µM |
| Benproperine | Selleckchem | S5256 | 1 µM |
| Domperidone | Selleckchem | S2461 | 1 µM |
| Promethazine | Selleckchem | S5196 | 1 µM |
| Boldine | Selleckchem | S9050 | 1 µM |
| CK-666 | Selleckchem | S7690 | 0.1-3 µM |
| Bioactive Compound Library | Selleckchem |  |  |
| Immunocytochemistry | | | |
| PFA | Thermo Scientific | 11586711 | 4% |
| Triton | Sigma | T8787 | 0.05% |
| BSA | Sigma | A9647 | 1% |
| Antibodies | | | |
| Anti-Ki67 (rabbit) | Abcam | ab16667 | 1:1000 |
| Anti-HUCD (mouse) | Invitrogen | A21271 | 1:1000 |
| Anti-MAP2 | Novus | NB300-213 | 1:5000 |
| Anti-Synapsin 1 | Synaptic System | 106011 | 1:500 |
| Anti-SHANK3 | Synaptic System | 162304 | 1:1000 |
| Anti-SHANK3 (for Western blotting) | MyBioSource | MBS150144 | 1 μg/ml |
| Anti-ARPC2 | Abcam | ab133315 | 1:500 |
| Anti-β-actin | CellSignalling | 3700 | 1:500 |
| Anti-SSEA-4-PE | Miltenyi Biotec | 130-122-914 | 1:50 |
| REAffinity control-PE | Miltenyi Biotec | 130-113-462 | 1:50 |
| Anti-SSEA-3-PE | Biolegend | 330312 | 1:50 |
| REAffinity control-PE | Miltenyi Biotec | 130-113-462 | 1:50 |
| Anti-TRA-1-81-AF647 | Biolegend | 330706 | 1:25 |
| Isotype Alexa Fluor 647 Mouse | Biolegend | 401618 | 1:25 |
| Anti-rabbit AF 647 | Invitrogen | A21245 | 1:1000 |
| Anti-mouse AF 488 | Invitrogen | A11029 | 1:1000 |
| Anti-chicken AF 555 | Jackson ImmunoResearch | JIR703-585-155 | 1:250 |
| Anti-guinea pig AF 647 | Jackson ImmunoResearch | JIR706-605-148 | 1:2000 |
| Hoechst | Mol.probes | H3570 | 1:2500 |
| Cell lines | | | |
| Name | **SHANK3 exon 21 mutation** | | **Source** |
| ASD01 | E809X | | (18) |
| ASD03 | G1271Afs*15 | | (18) |
| ASD04 | L1142Vfs*153 | | (18) |
| PDF01 | None detected | | ATCC |
| 4603 | None detected | | Coriell, (20) |
| PC056 | None detected | | Phenocell |
| SA001 clone WT1 | Guide transfected, no editing detected | | (19) |
| SA001 clone WT2 | Guide transfected, no editing detected | | (19) |
| SA001 clone WT3 | Guide transfected, no editing detected | | (19) |
| SA001 clone HT1 | c.2401_2426del (single allele) | | (19) |
| SA001 clone HT2 | c.2401_2407del (single allele) | | (19) |
| SA001 clone HT3 | c.2380_2402del (single allele) | | (19) |
| SA001 clone HM1 | c. 2380_2402del (both alleles) | | (19) |
| SA001 clone HM2 | c. 2380_2402del (both alleles) | | (19) |
