# Supplementary material for: Pharmacological modulation of developmental and synaptic phenotypes in human SHANK3 deficient stem cell-derived neuronal models
Source: Transl Psychiatry. 2024 Jun 10;14:249. doi: 10.1038/s41398-024-02947-3 (PMC11165012; doi:10.1038/s41398-024-02947-3)
Supplement: Supplementary file 4 — Table S3 [file 41398_2024_2947_MOESM4_ESM.docx]

|  | Compound name | CAS number | Target | Detailed annotation (Selleckchem) | Gene names (STRING input) | Pathway |
| --- | --- | --- | --- | --- | --- | --- |
| 1 | A-205804 | 251992-66-2 | Integrin | Inhibitor of E-selectin and ICAM-1 expression with IC50 of 20 nM and 25 nM respectively. | SELE, ICAM1 | Cytoskeletal Signaling |
| 2 | AS2863619 | 2241300-51-4 | CDK | Cyclin-dependent kinase CDK8/19 inhibitor with IC50 of 0.6099 nM and 4.277 nM, respectively. | CDK8, CDK9 | Cell Cycle |
| 3 | AZD1080 | 612487-72-6 | GSK-3 | Selective, orally active, brain permeable GSK3 inhibitor, inhibits human GSK3α and GSK3β with K_i_ of 6.9 nM and 31 nM, respectively. | GSK3A, GSK3B | PI3K/Akt/mTOR |
| 4 | AZD2858 | 486424-20-8 | GSK-3 | Selective GSK-3 inhibitor with an IC50 of 68 nM. | GSK3A, GSK3B | PI3K/Akt/mTOR |
| 5 | AZD5363 | 1143532-39-1 | Akt | Inhibits all isoforms of Akt(Akt1/Akt2/Akt3) with IC50 of 3 nM/8 nM/8 nM and lower activity towards ROCK1/2. | AKT1, AKT2, AKT3 | PI3K/Akt/mTOR |
| 6 | AZD5438 | 602306-29-6 | CDK | Inhibitor of CDK1/2/9 with IC50 of 16 nM/6 nM/20 nM in cell-free assays. It also inhibits GSK3β. | CDK1, CDK2, CDK9, GSK3B | Cell Cycle |
| 7 | Alimemazine Tartrate | 4330-99-8 | Others | Phenothiazine derivative that is used as an antipruritic. |  | Others |
| 8 | Autophinib | 1644443-47-9 | Autophagy,PI3K | Inhibitor with IC50 values of 90 and 40 nM for autophagy in starvation induced autophagy assay and rapamycin induced autophagy assay. The IC50 value for Vps34 is 19 nM in vitro. | PIK3C3 | Autophagy |
| 9 | BI-1347 | 2163056-91-3 | CDK | Inhibitor of CDK8 with IC50 of 1.1 nM. | CDK8 | Cell Cycle |
| 10 | BMS-265246 | 582315-72-8 | CDK | CDK1/2 inhibitor with IC50 of 6 nM/9 nM in a cell-free assay. | CDK1, CDK2 | Cell Cycle |
| 11 | Benproperine phosphate | 19428-14-9 | Others | Cough suppressant. Is an orally active, potent actin-related protein 2/3 complex subunit 2 (ARPC2) inhibitor. Attenuates actin polymerization. | ARPC2 | Others |
| 12 | Boldine | 476-70-0 | FXR receptor | Isolated from Peumus boldus, has alpha-adrenergic antagonist. | NR1H4 | Others |
| 13 | CP21R7 (CP21) | 125314-13-8 | Wnt/beta-catenin | GSK-3β inhibitor that can potently activate canonical Wnt signalling. | GSK3B | Stem Cells & Wnt |
| 14 | Cepharanthine | 481-49-2 | TNF-alpha | Inhibits tumor necrosis factor TNFα-mediated NFκB stimulation | TNF | Apoptosis |
| 15 | Cerdulatinib (PRT062070, PRT2070) | 1369761-01-2 | JAK | Tyrosine kinase inhibitor with IC50 of 12 nM/6 nM/8 nM/0.5 nM and 32 nM for JAK1/JAK2/JAK3/TYK2 and Syk, respectively. | JAK1, JAK2, JAK3, TYK2, SYK | JAK/STAT |
| 16 | Domperidone | 57808-66-9 | Dopamine Receptor | Oral dopamine D2 receptor antagonist, used to treat nausea and vomiting. | DRD2 | Neuronal Signaling |
| 17 | Doramapimod (BIRB 796) | 285983-48-4 | p38 MAPK | Pan-p38 MAPK inhibitor with IC50 of 38 nM, 65 nM, 200 nM and 520 nM for p38α/β/γ/δ. | MAPK11, MAPK12, MAPK13,MAPK14 | MAPK |
| 18 | GO-203 | 1222186-26-6 | Others | Peptide inhibitor of MUC1-C dimerization. | MUC1 | Others |
| 19 | GSK269962A HCl | 850664-21-0 | ROCK | Selective ROCK inhibitor with IC50 values of 1.6 and 4 nM for ROCK1 and ROCK2, respectively | ROCK1, ROCK2 | Cell Cycle |
| 20 | GSK429286A | 864082-47-3 | ROCK | Selective inhibitor of ROCK1 and ROCK2 with IC50 of 14 nM and 63 nM, respectively. | ROCK1, ROCK2 | Cell Cycle |
| 21 | Geneticin (G418 Sulfate) | 108321-42-2 | Anti-infection | Elongation inhibitor of the 80S ribosome. |  | Microbiology |
| 22 | Hexachlorophene | 70-30-4 | Potassium Channel | KCNQ1/KCNE1 potassium channel activator with EC50 of 4.61 ± 1.29 μM; It can also attenuate Wnt/beta-catenin signaling. | KCNQ1, KCNE1 | Transmembrane Transporters |
| 23 | Isotretinoin | 4759-48-2 | Hydroxylase | Developed as chemotherapy medication against brain & pancreatic cancer. |  | Metabolism |
| 24 | LY2090314 | 603288-22-8 | GSK-3 | GSK-3 inhibitor for GSK-3α/β with IC50 of 1.5 nM/0.9 nM. | GSK3A, GSK3B | PI3K/Akt/mTOR |
| 25 | Lycorine | 476-28-8 | AChR | Inhibits acetylcholinesterase (AChE) and ascorbic acid biosynthesis. | ACHE | Neuronal Signaling |
| 26 | Lycorine hydrochloride | 2188-68-3 | HCV Protease | HCV inhibitor. |  | Viral Proteases |
| 27 | MSC2530818 | 1883423-59-3 | CDK | CDK8 inhibitor with the IC50 of 2.6 nM. | CDK8 | Cell Cycle |
| 28 | Momelotinib (CYT387) | 1056634-68-4 | JAK | Inhibitor of JAK1/JAK2 with IC50 of 11 nM/18 nM. | JAK1, JAK2 | JAK/STAT |
| 29 | ORY-1001 (RG-6016) 2HCl | 1431326-61-2 | Histone Demethylase | Lysine-specific demethylase KDM1A inhibitor with IC50 of <20 nM. | LSD1 | Epigenetics |
| 30 | PHA-767491 | 942425-68-5 | CDK | Cdc7/CDK9 inhibitor with IC50 of 10 nM and 34 nM in cell-free assays, respectively. | CDC7, CDK9 | Cell Cycle |
| 31 | Pexmetinib (ARRY-614) | 945614-12-0 | p38 MAPK,Tie-2 | Dual p38 MAPK/Tie-2 inhibitor with IC50 of 4 nM/18 nM. | MAPK11, MAPK12, MAPK13,MAPK14, TEK | MAPK |
| 32 | Promethazine HCl | 58-33-3 | Histamine Receptor | Histamine H_1_ receptor antagonist, used as a sedative and antiallergic medication. | HRH1 | Neuronal Signaling |
| 33 | RKI-1447 | 1342278-01-6 | ROCK | Inhibitor of ROCK1 and ROCK2, with IC50 of 14.5 nM and 6.2 nM, respectively. | ROCK1, ROCK2 | Cell Cycle |
| 34 | RepSox | 446859-33-2 | TGF-beta/Smad | Inhibitor of the TGFβR-1 with IC50 of 23 nM. | TGFBR1 | TGF-beta/Smad |
| 35 | SR-4370 | 1816294-67-3 | HDAC | Inhibitor of class I HDACs with IC50 of 0.13 µM, 0.58 µM, 0.006 µM, 2.3 µM, 3.7 µM for HDAC 1, HDAC 2, HDAC 3, HDAC 8, HDAC 6, respectively. | HDAC1, HDAC3 | Epigenetics |
| 36 | Skepinone-L | 1221485-83-1 | p38 MAPK | p38α-MAPK inhibitor with IC50 of 5 nM. | MAPK14 | MAPK |
| 37 | TP0427736 HCl | 864374-00-5 | ALK | Inhibitor of TGFβR-1 with an IC50 of 2.72 nM. | TGFBR1 | Angiogenesis |
| 38 | Tretinoin | 302-79-4 | Retinoid Receptor | Ligand for both the retinoic acid receptor (RAR) and the retinoid X receptor (RXR). | RARA, RARB, RARG, RXRA, RXRB, RXRG | Metabolism |
| 39 | Trimipramine Maleate | 521-78-8 | Others | Antidepressant; serotonin transport blocker that also blocks norepinephrine uptake. |  | Others |
| 40 | UM171 | 1448724-09-1 | Others |  |  | Others |
| 41 | WS6 | 1421227-53-3 | IκB/IKK | Cell proliferation inducer via modulation of Erb3 binding protein-1 (EBP1) and the IκB kinase pathway. | PA2G4, IKBKB | NF-κB |
| 42 | Y-39983 HCl | 173897-44-4 | ROCK | Selective rho-associated protein kinase(ROCK) inhibitor with an IC50 of 3.6 nM. | ROCK1, ROCK2 | Cell Cycle |
