# Supplementary material for: Pharmacological modulation of developmental and synaptic phenotypes in human SHANK3 deficient stem cell-derived neuronal models
Source: Transl Psychiatry. 2024 Jun 10;14:249. doi: 10.1038/s41398-024-02947-3 (PMC11165012; doi:10.1038/s41398-024-02947-3)
Supplement: Supplementary file 5 — Table S4 [file 41398_2024_2947_MOESM5_ESM.docx]

| iPSC lines | | | | | |
| --- | --- | --- | --- | --- | --- |
| Cell line name | **SHANK3 mutation type** | | **Genetic abnormality** | **Inheritance** | **Sex** |
| ASD01 | SHANK3 STOP truncation | E809X | | de novo | F |
| ASD03 | SHANK3 frame shift truncation | G1271Afs*15 | | de novo | M |
| ASD04 | SHANK3 frame shift truncation | L1142Vfs*153 | | de novo | M |
| PDF01 | None detected | None detected | | N/A | M |
| 4603 | None detected | None detected | | N/A | M |
| PC056 | None detected | None detected | | N/A | F |
| hESC lines (all clones derived from hESC line SA001) | | | | | |
| Cell line name | **Clone** | **Confirmed CRISPR editing (Shank3 Exon 21)** | | **Predicted effect on translation** | **Sex** |
| WT1 | SA01 g141 cl2 B04 | Guide transfected, no editing detected | | N/A | M |
| WT2 | SA01 g141 cl2 H07 | Guide transfected, no editing detected | | N/A | M |
| WT3 | SA01 g141 cl3 G04 | Guide transfected, no editing detected | | N/A | M |
| HT1 | SA01 g141 cl2 D04 | c.2401_2426del | | p.R802Qfs*18 (C-terminal deletion) | M |
| HT2 | SA01 g141 cl3 D05 | c.2401_2407del | | p.L801Qfs*54 (C-terminal deletion) | M |
| HT3 | SA01 g141 cl7 A02 | c.2380_2402del | | p.A796Rfs*25 (C-terminal deletion) | M |
| HM1 | SA01 g150 cl1 E07 | c. 2380_2402del (both alleles) | | p.L801Qfs*49 (C-terminal deletion) | M |
| HM2 | SA01 g150 cl1 C07 | c. 2380_2402del (both alleles) | | p.L801Qfs*49 (C-terminal deletion) | M |
