# Supplementary figures and images for: Pharmacological modulation of developmental and synaptic phenotypes in human SHANK3 deficient stem cell-derived neuronal models
Source: Transl Psychiatry. 2024 Jun 10;14:249. doi: 10.1038/s41398-024-02947-3 (PMC11165012; doi:10.1038/s41398-024-02947-3)

**A**

PMDS patient iPSC lines

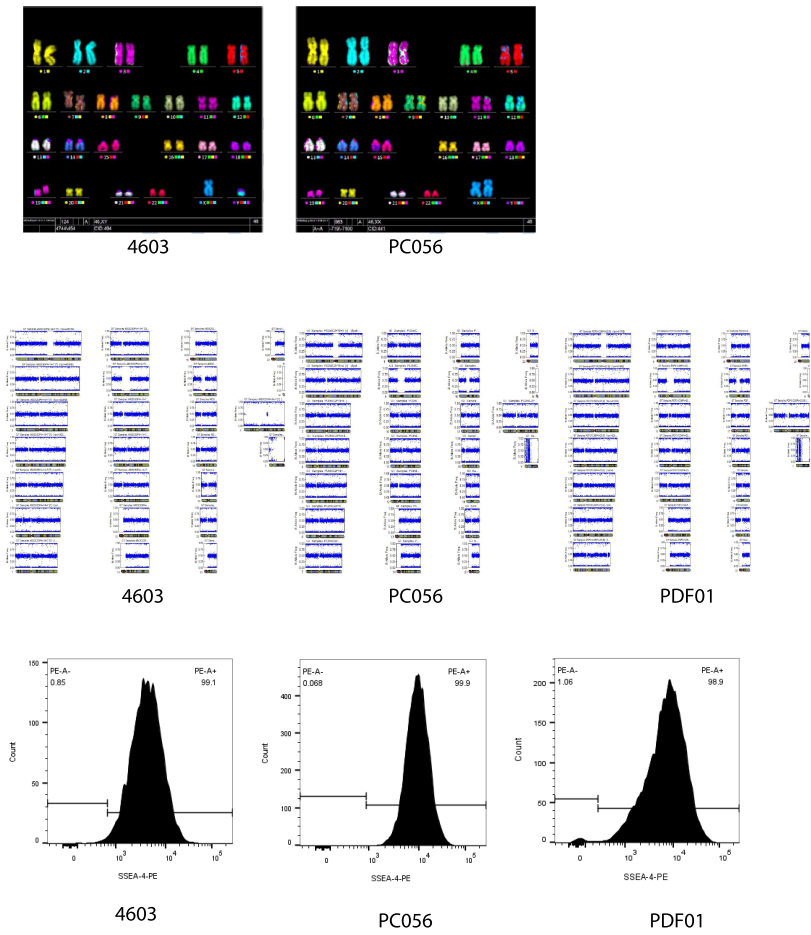

**B**

Healthy donor iPSC lines

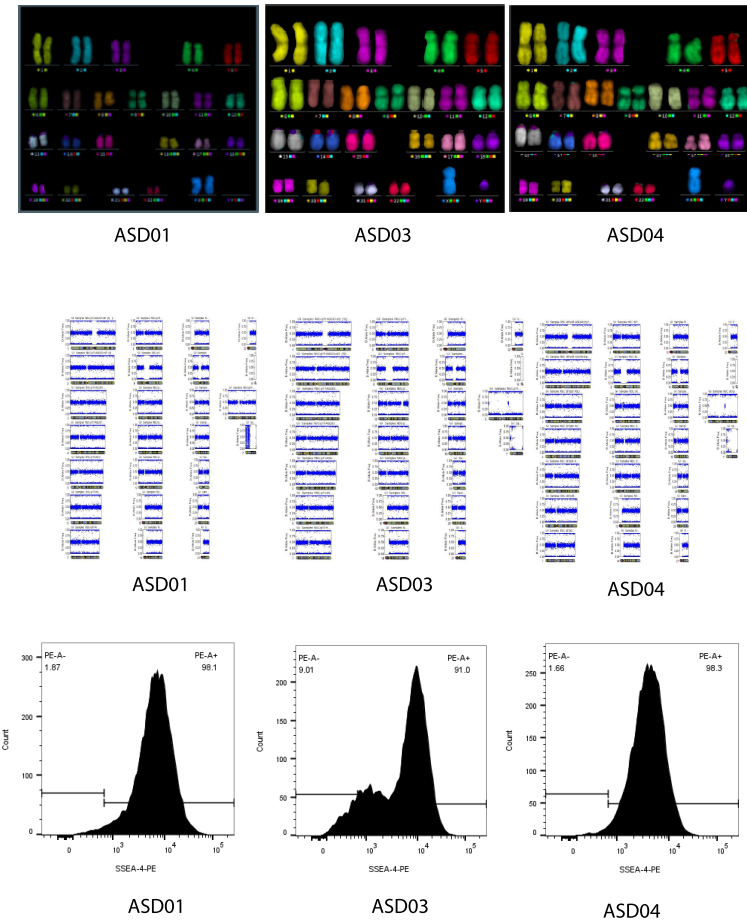

Supplement: Supplementary file 6 — Figure S1 [file 41398_2024_2947_MOESM6_ESM.pdf]

**A**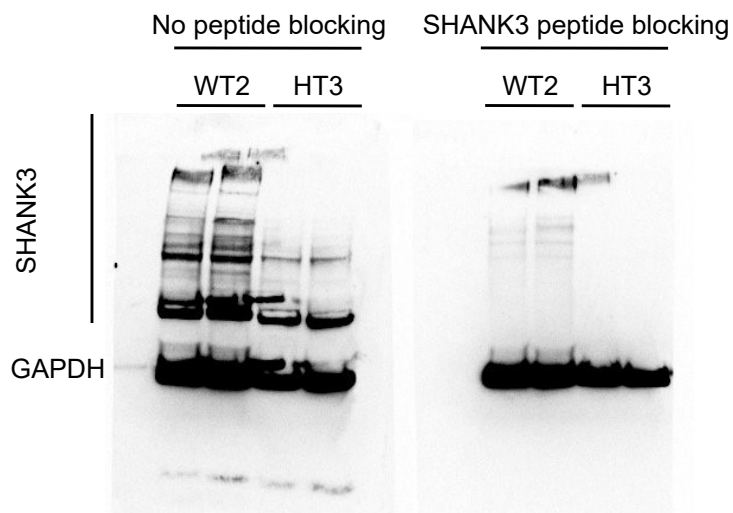**B**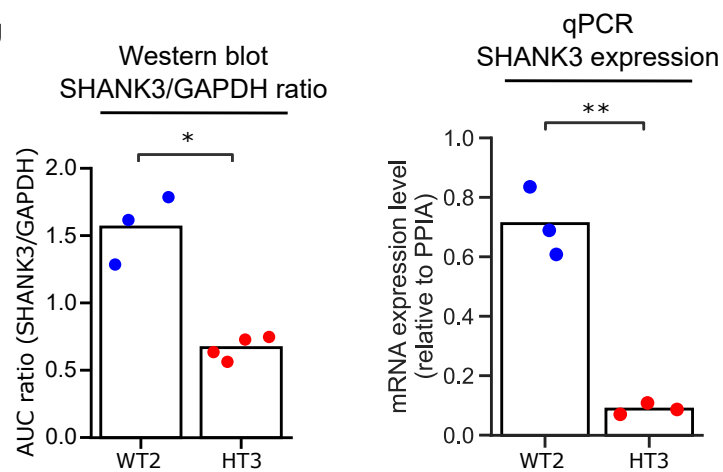**C**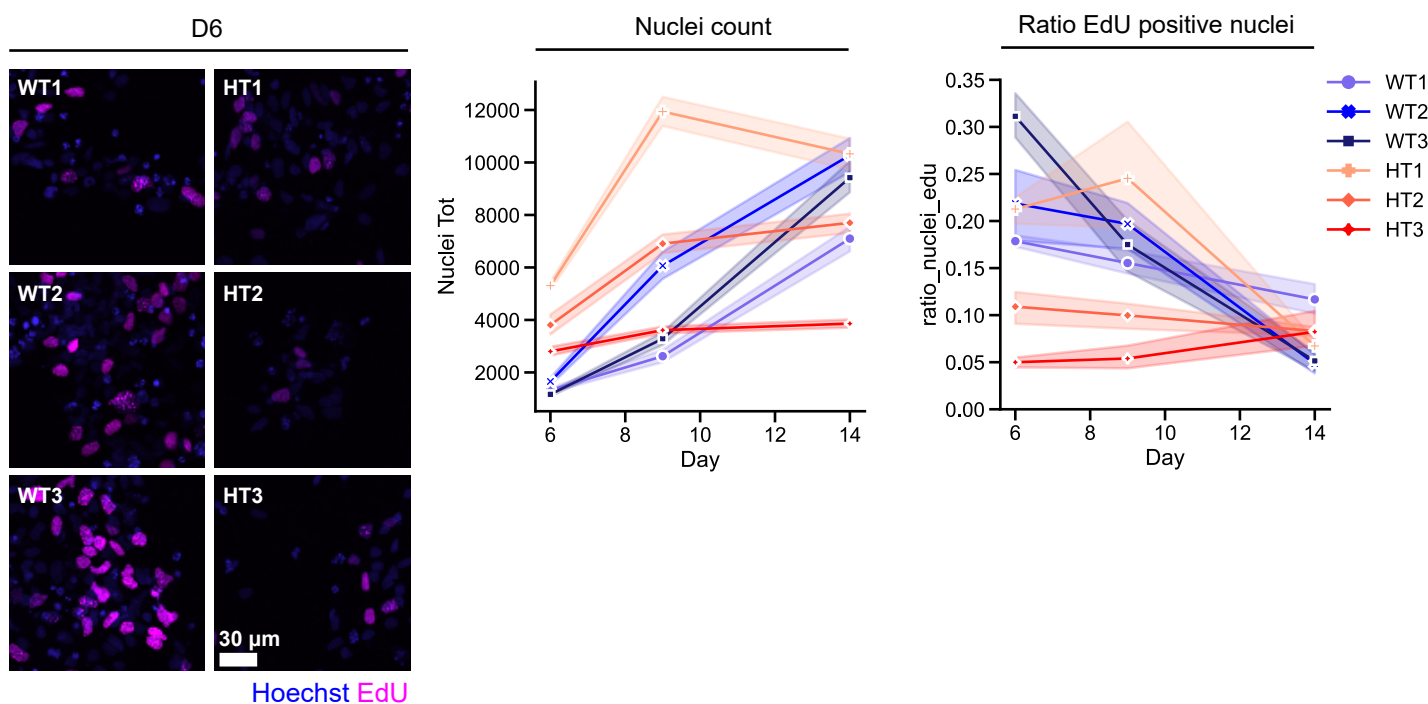**D**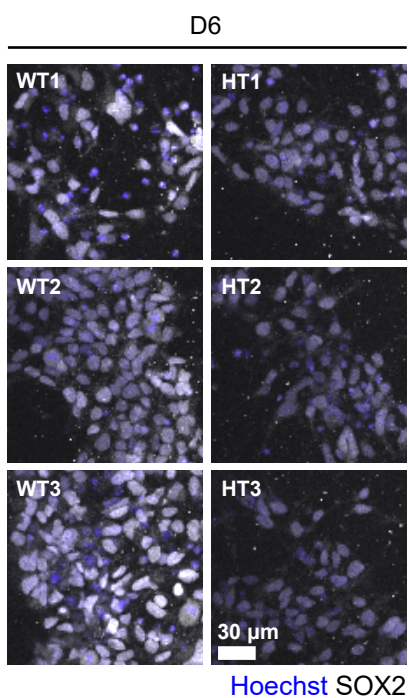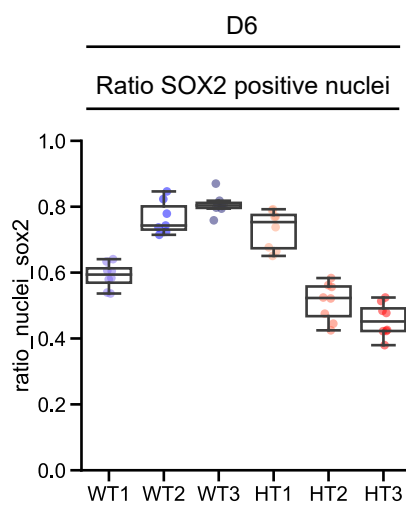**E**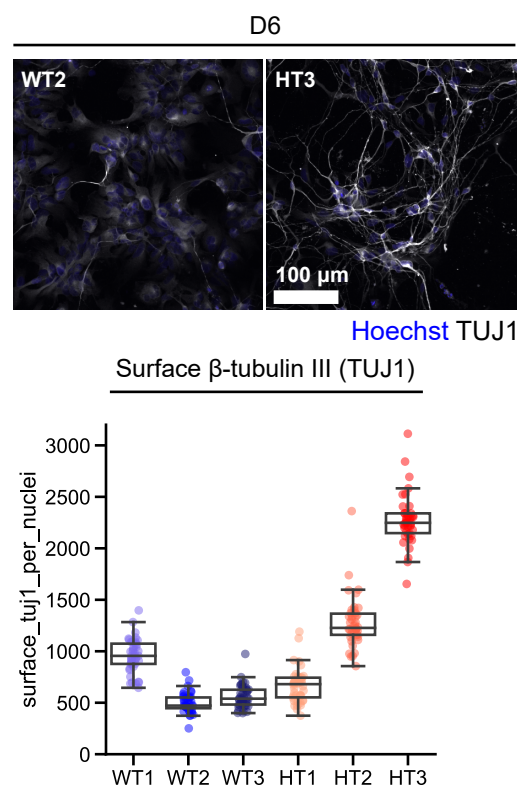

Supplement: Supplementary file 8 — Figure S3 [file 41398_2024_2947_MOESM8_ESM.pdf]

**A**

PMDS patient-derived NPCs (Figure 1C data pooled)

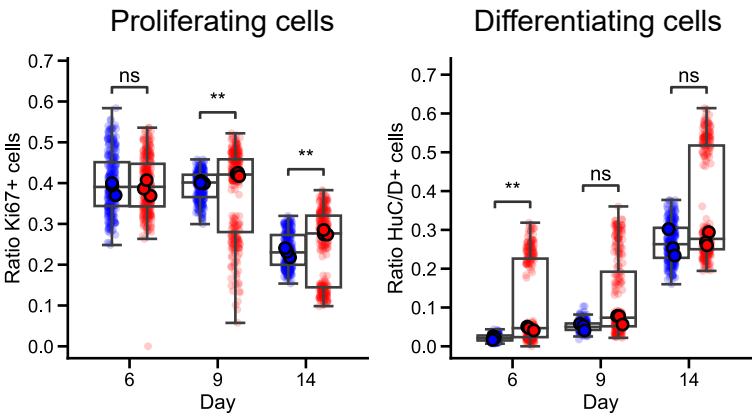

**B**

CRISPR-engineered NPCs (Figure 1F data pooled)

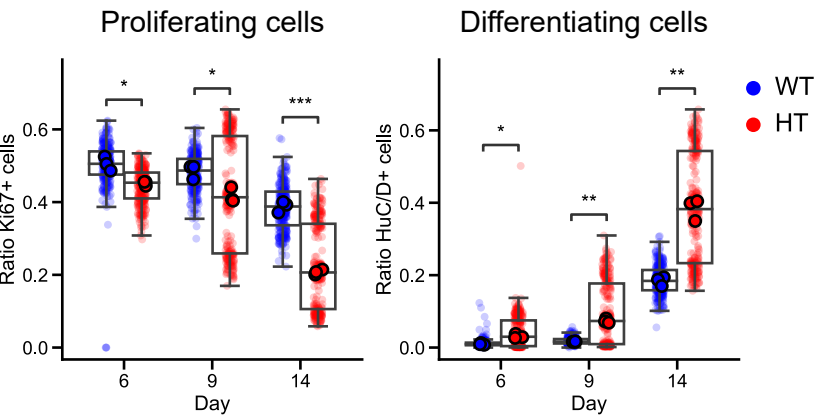

Supplement: Supplementary file 9 — Figure S4 [file 41398_2024_2947_MOESM9_ESM.pdf]

A

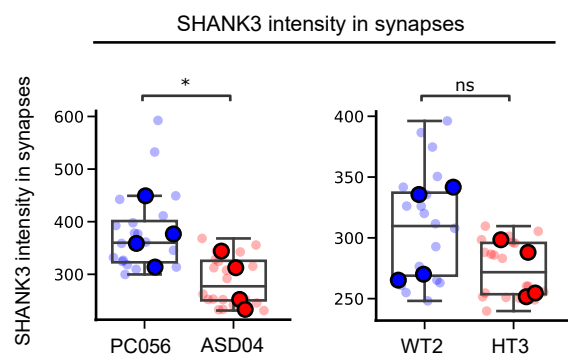

Supplement: Supplementary file 10 — Figure S5 [file 41398_2024_2947_MOESM10_ESM.pdf]

**A**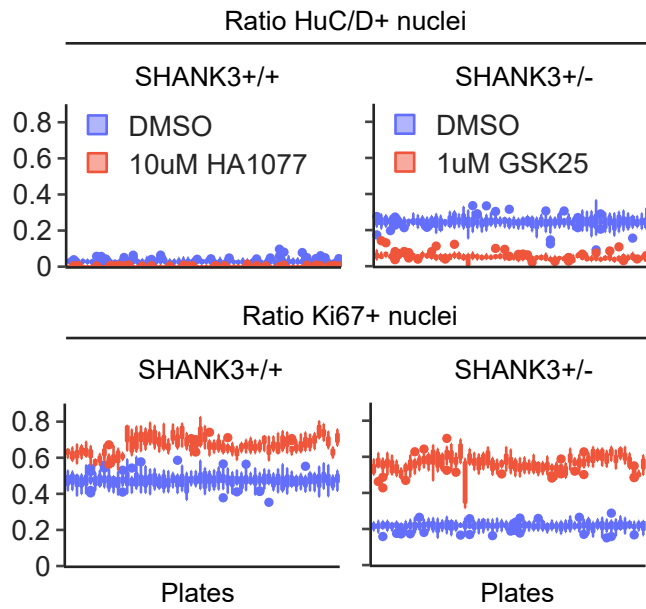**B**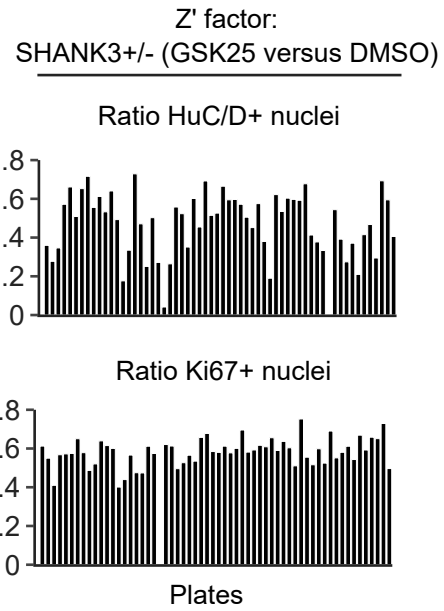**C**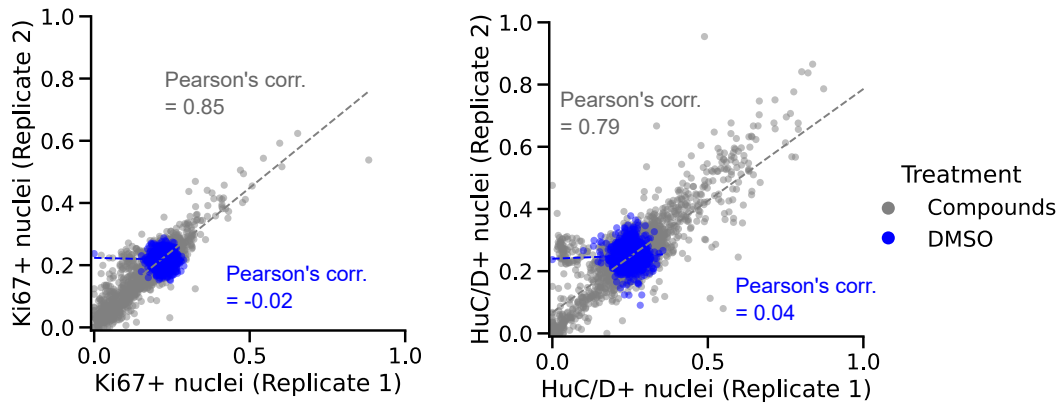

Supplement: Supplementary file 11 — Figure S6 [file 41398_2024_2947_MOESM11_ESM.pdf]

**A**

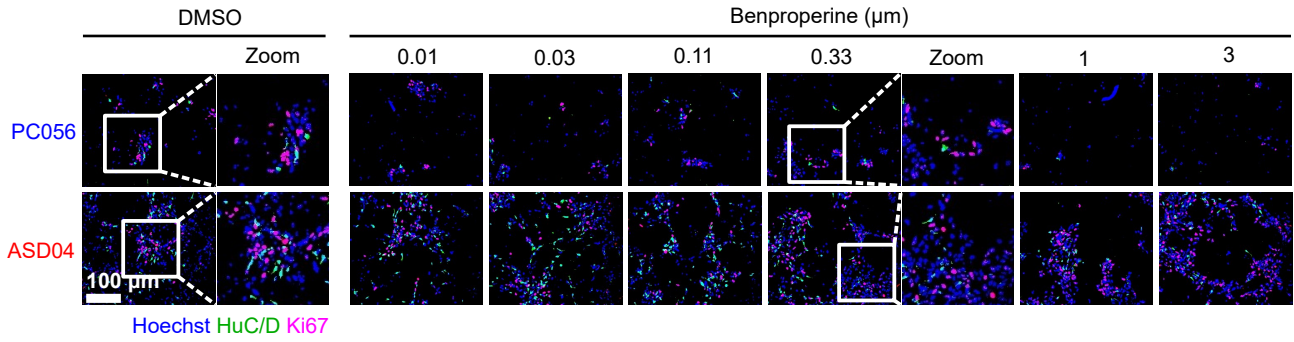

**B**

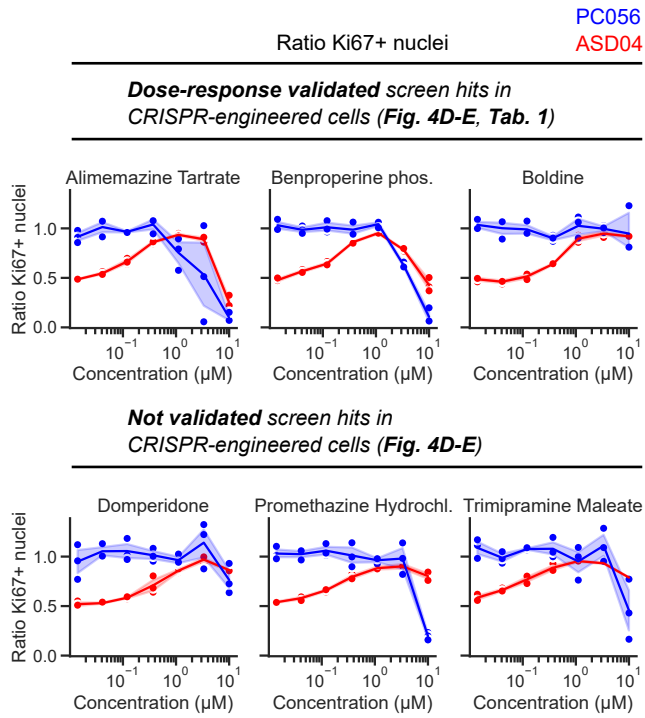

**C**

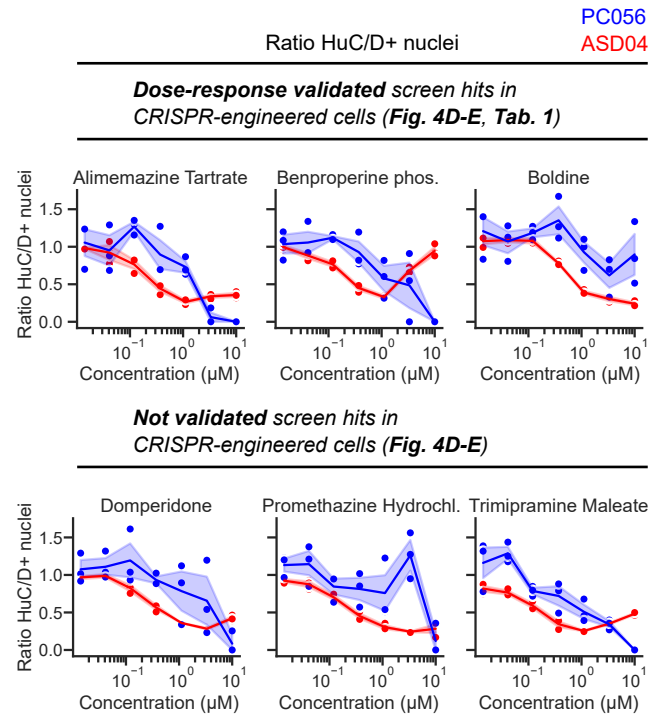

Supplement: Supplementary file 12 — Figure S7 [file 41398_2024_2947_MOESM12_ESM.pdf]

**A**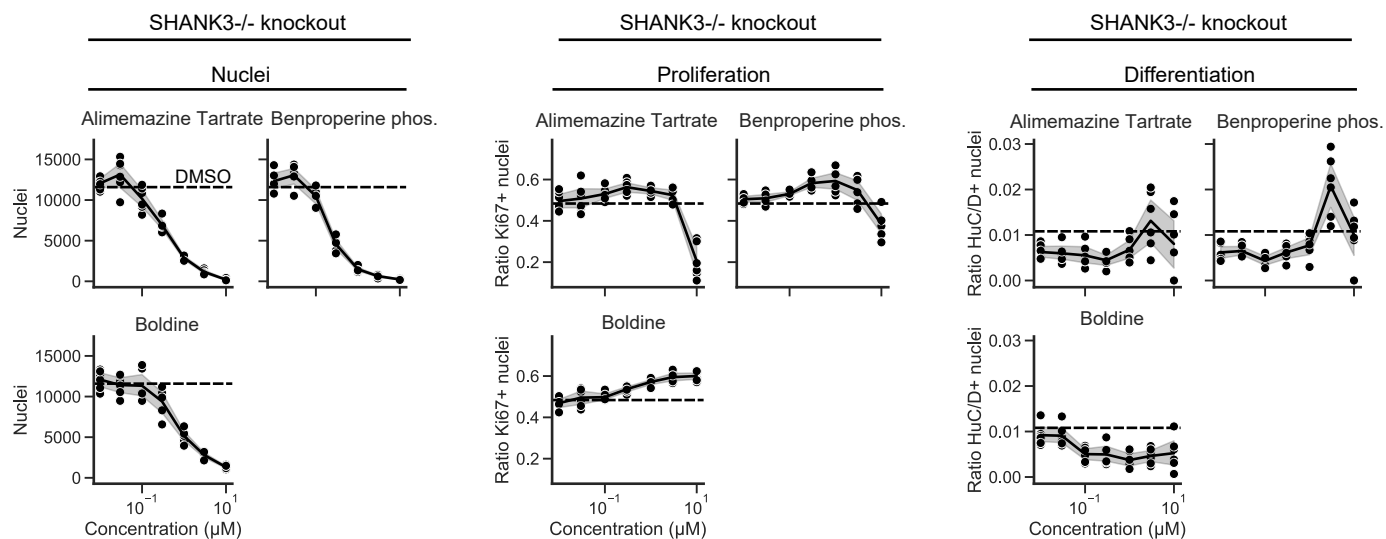**B**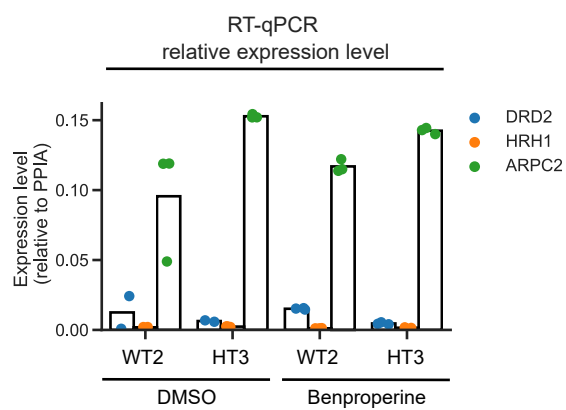

Supplement: Supplementary file 13 — Figure S8 [file 41398_2024_2947_MOESM13_ESM.pdf]

A

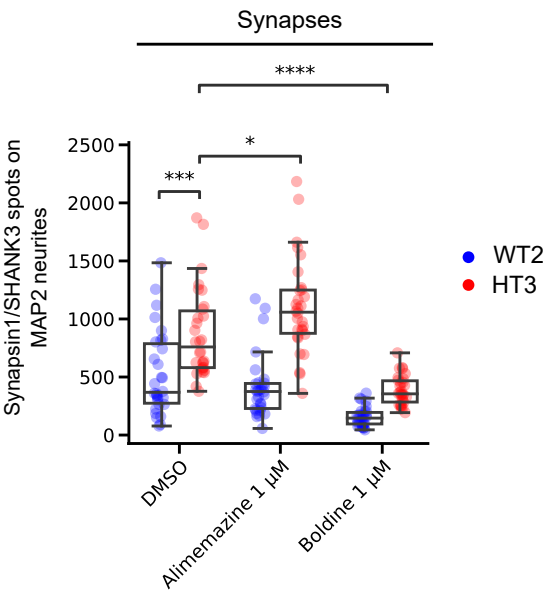

Supplement: Supplementary file 14 — Figure S9 [file 41398_2024_2947_MOESM14_ESM.pdf]
