# Supplementary material for: Pharmacological modulation of developmental and synaptic phenotypes in human SHANK3 deficient stem cell-derived neuronal models
Source: Transl Psychiatry. 2024 Jun 10;14:249. doi: 10.1038/s41398-024-02947-3 (PMC11165012; doi:10.1038/s41398-024-02947-3)
Supplement: Supplementary file 7 — Figure S2 [file 41398_2024_2947_MOESM7_ESM.pdf]

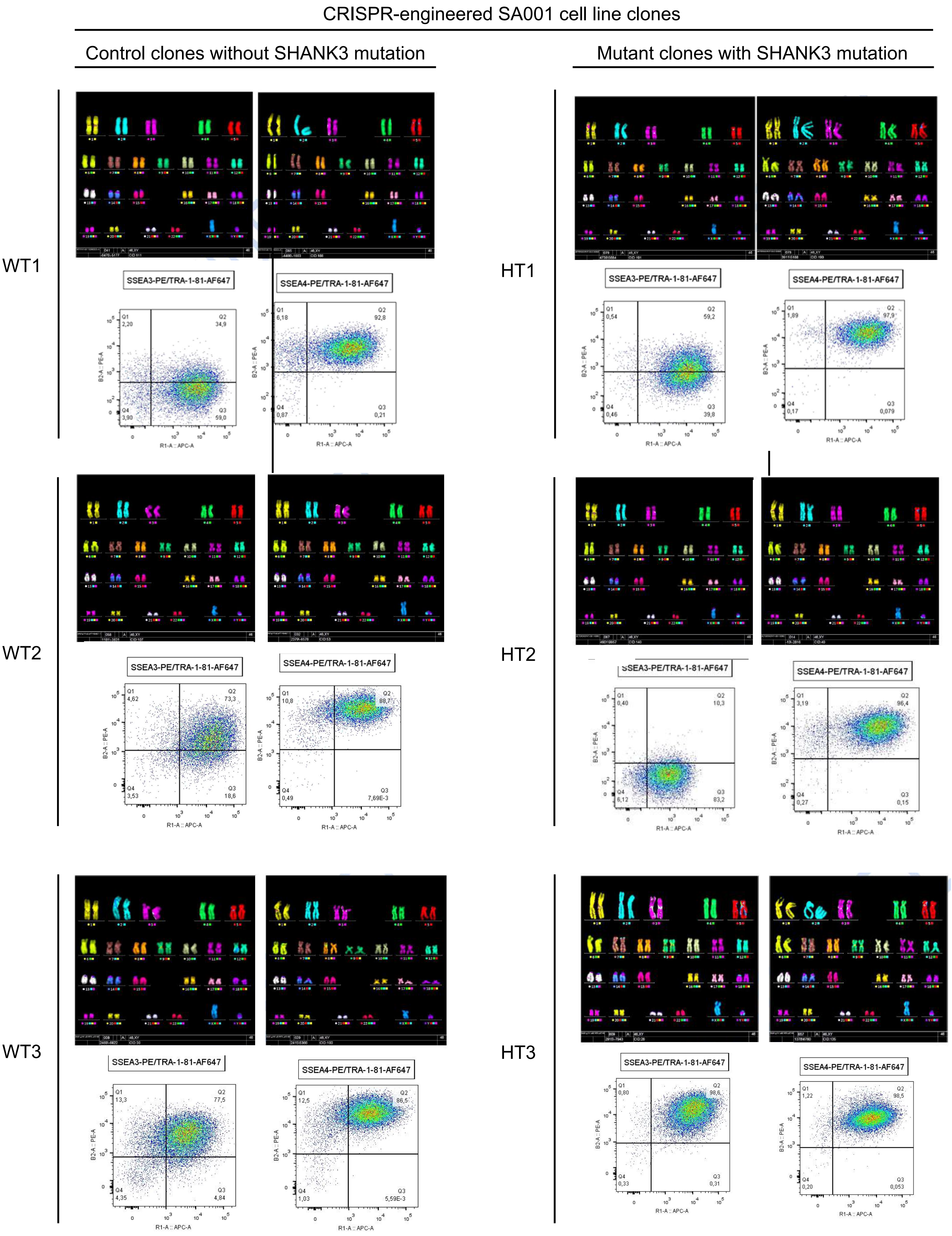
CRISPR-engineered SA001 cell line clones
Control clones without SHANK3 mutation
Mutant clones with SHANK3 mutation
WT1
SSEA3-PE/TRA-1-81-AF647
Q1 2,20
Q2 34,9
Q4 3,90
Q3 59,0
SSEA4-PE/TRA-1-81-AF647
Q1 6,18
Q2 92,8
Q4 0,87
Q3 0,21
WT2
SSEA3-PE/TRA-1-81-AF647
Q1 4,62
Q2 73,3
Q4 3,53
Q3 18,6
SSEA4-PE/TRA-1-81-AF647
Q1 10,8
Q2 88,7
Q4 0,49
Q3 7,69E-3
WT3
SSEA3-PE/TRA-1-81-AF647
Q1 13,3
Q2 77,5
Q4 4,35
Q3 4,84
SSEA4-PE/TRA-1-81-AF647
Q1 12,5
Q2 86,5
Q4 1,03
Q3 5,59E-3
HT1
SSEA3-PE/TRA-1-81-AF647
Q1 0,54
Q2 59,2
Q4 0,46
Q3 39,8
SSEA4-PE/TRA-1-81-AF647
Q1 1,89
Q2 97,9
Q4 0,17
Q3 0,079
HT2
SSEA3-PE/TRA-1-81-AF647
Q1 0,40
Q2 10,3
Q4 6,12
Q3 83,2
SSEA4-PE/TRA-1-81-AF647
Q1 3,19
Q2 96,4
Q4 0,27
Q3 0,15
HT3
SSEA3-PE/TRA-1-81-AF647
Q1 0,80
Q2 98,6
Q4 0,33
Q3 0,31
SSEA4-PE/TRA-1-81-AF647
Q1 1,22
Q2 98,5
Q4 0,20
Q3 0,053
B2-A :: PE-A
R1-A :: APC-A
