# Supplementary material for: Pharmacological modulation of developmental and synaptic phenotypes in human SHANK3 deficient stem cell-derived neuronal models
Source: Transl Psychiatry. 2024 Jun 10;14:249. doi: 10.1038/s41398-024-02947-3 (PMC11165012; doi:10.1038/s41398-024-02947-3)
Supplement: Supplementary file 15 — Figure S10 [file 41398_2024_2947_MOESM15_ESM.pdf]

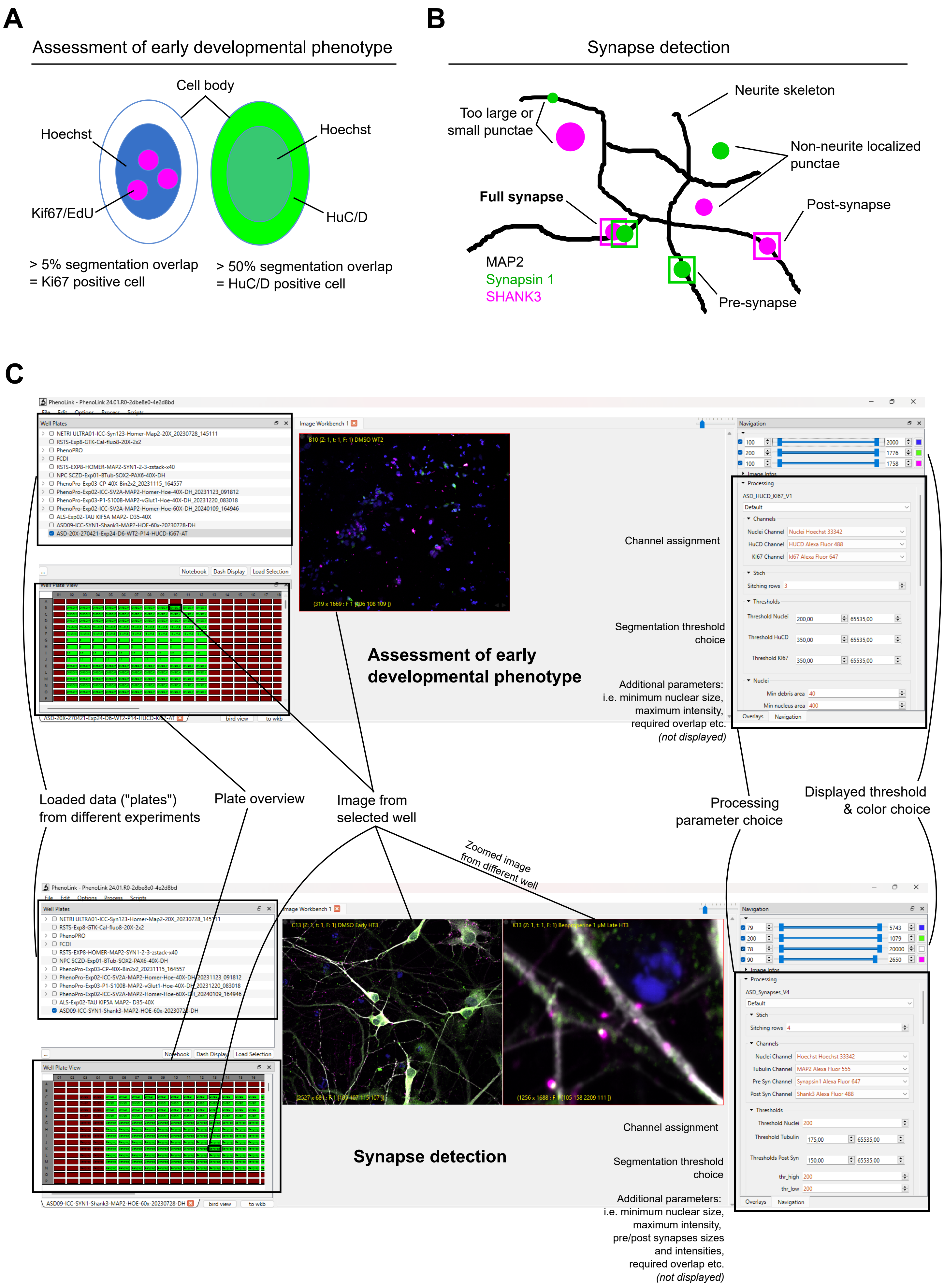

A
Assessment of early developmental phenotype
Cell body
Hoechst
Hoechst
Kif67/EdU
HuC/D
> 5% segmentation overlap
= Ki67 positive cell
> 50% segmentation overlap
= HuC/D positive cell
B
Synapse detection
Neurite skeleton
Too large or
small punctae
Non-neurite localized
punctae
Full synapse
Post-synapse
MAP2
Synapsin 1
SHANK3
Pre-synapse
C
Channel assignment
Segmentation threshold
choice
Assessment of early
developmental phenotype
Additional parameters:
i.e. minimum nuclear size,
maximum intensity,
required overlap etc.
(not displayed)
Loaded data ("plates")
from different experiments
Plate overview
Image from
selected well
Processing
parameter choice
Displayed threshold
& color choice
Zoomed image
from different well
Synapse detection
Channel assignment
Segmentation threshold
choice
Additional parameters:
i.e. minimum nuclear size,
maximum intensity,
pre/post synapses sizes
and intensities,
required overlap etc.
(not displayed)
